# Supplementary material for: “As it is about that, they do as they please”: Women’s experience of the accessibility and acceptability of postabortion care in Kaya, Burkina Faso
Source: PLOS Glob Public Health. 2026 Jan 8;6(1):e0005035. doi: 10.1371/journal.pgph.0005035 (PMC12782387; doi:10.1371/journal.pgph.0005035)
Supplement: S1 File — (DOCX) [file pgph.0005035.s001.docx]

**S1 File: Reporting checklist for qualitative study.**

Based on the Standards for Reporting Qualitative Research (SRQR) guideline* for the article:

"*As it is about that, they do as they please*": Women’s experience of the accessibility and acceptability of postabortion care in Kaya, Burkina Faso.

| **Reporting item** | | **Page/line no(s).** |
| --- | --- | --- |
| **Title and abstract** | | |
|  | **Title** - Concise description of the nature and topic of the study Identifying the study as qualitative or indicating the approach (e.g., ethnography, grounded theory) or data collection methods (e.g., interview, focus group) is recommended | Page 1 |
|  | **Abstract** - Summary of key elements of the study using the abstract format of the intended publication; typically includes background, purpose, methods, results, and conclusions | Page 2-3  Lines 23-52 |
| **Introduction** | | |
|  | **Problem formulation** - Description and significance of the problem/phenomenon studied; review of relevant theory and empirical work; problem statement | Pages 4-8  Lines 76-157 |
|  | **Purpose or research question** - Purpose of the study and specific objectives or questions | Page 7  Lines 158-173 |
| **Methods** | | |
|  | **Qualitative approach and research paradigm** - Qualitative approach (e.g., ethnography, grounded theory, case study, phenomenology, narrative research) and guiding theory if appropriate; identifying the research paradigm (e.g., postpositivist, constructivist/ interpretivist) is also recommended; rationale** | Page 9; 12-14  Lines 197-199; 266-309 |
|  | **Researcher characteristics and reflexivity** - Researchers’ characteristics that may influence the research, including personal attributes, qualifications/experience, relationship with participants, assumptions, and/or presuppositions; potential or actual interaction between researchers’ characteristics and the research questions, approach, methods, results, and/or transferability | Page 12  Lines 298-302 |
|  | **Context** - Setting/site and salient contextual factors; rationale** | Pages 6-7; 8-9  Lines 123-125; 175-195 |
|  | **Sampling strategy** - How and why research participants, documents, or events were selected; criteria for deciding when no further sampling was necessary (e.g., sampling saturation); rationale** | Pages 9-10  Lines 197-221 |
|  | **Ethical issues pertaining to human subjects** - Documentation of approval by an appropriate ethics review board and participant consent, or explanation for lack thereof; other confidentiality and data security issues | Page 14  Lines 310-319 |
|  | **Data collection methods** - Types of data collected; details of data collection procedures including (as appropriate) start and stop dates of data collection and analysis, iterative process, triangulation of sources/methods, and modification of procedures in response to evolving study findings; rationale** | Pages 10-11  Lines 222-264 |
|  | **Data collection instruments and technologies** - Description of instruments (e.g., interview guides, questionnaires) and devices (e.g., audio recorders) used for data collection; if/how the instrument(s) changed over the course of the study | Pages 11-12  Lines 233-249, 250-258 |
|  | **Units of study** - Number and relevant characteristics of participants, documents, or events included in the study; level of participation (could be reported in results) | Pages 10; 11  Lines 223-225; 250-251 |
|  | **Data processing** - Methods for processing data prior to and during analysis, including transcription, data entry, data management and security, verification of data integrity, data coding, and anonymization/de-identification of excerpts | Page 12  Lines 259-264 |
|  | **Data analysis** - Process by which inferences, themes, etc., were identified and developed, including the researchers involved in data analysis; usually references a specific paradigm or approach; rationale** | Pages 12-14  Lines 265-309 |
|  | **Techniques to enhance trustworthiness** - Techniques to enhance trustworthiness and credibility of data analysis (e.g., member checking, audit trail, triangulation); rationale** | Page 13  Lines 298-302 |
| **Results** | | |
|  | **Synthesis and interpretation** - Main findings (e.g., interpretations, inferences, and themes); might include development of a theory or model, or integration with prior research or theory | Pages 14-33  Lines 320-728 |
|  | **Links to empirical data** - Evidence (e.g., quotes, field notes, text excerpts, photographs) to substantiate analytic findings | Pages 14-33  Lines 320-728 |
| **Discussion** | | |
|  | **Integration with prior work, implications, transferability, and contribution(s) to the field** - Short summary of main findings; explanation of how findings and conclusions connect to, support, elaborate on, or challenge conclusions of earlier scholarship; discussion of scope of application/generalizability; identification of unique contribution(s) to scholarship in a discipline or field | Pages 33- 40  Lines 731-845 |
|  | **Limitations** - Trustworthiness and limitations of findings | Pages 38-39  Lines 846-878 |
| **Other** | | |
|  | **Conflicts of interest** - Potential sources of influence or perceived influence on study conduct and conclusions; how these were managed | Page 41  Lines 910-911 |
|  | **Funding** - Sources of funding and other support; role of funders in data collection, interpretation, and reporting | Page 40-41  Lines 907-910 |

*O'Brien BC, Harris IB, Beckman TJ, Reed DA, Cook DA. Standards for reporting qualitative research: a synthesis of recommendations. Academic Medicine, Vol. 89, No. 9 / Sept 2014

DOI: 10.1097/ACM.0000000000000388
