# Supplementary material for: “As it is about that, they do as they please”: Women’s experience of the accessibility and acceptability of postabortion care in Kaya, Burkina Faso
Source: PLOS Glob Public Health. 2026 Jan 8;6(1):e0005035. doi: 10.1371/journal.pgph.0005035 (PMC12782387; doi:10.1371/journal.pgph.0005035)
Supplement: S1 Checklist — (DOCX) [file pgph.0005035.s002.docx]

Inclusivity in global research

PLOS’ policy on inclusivity in global research aims to improve transparency in the reporting of research performed outside of researchers’ own country or community and ensures that PLOS publications reporting global research adhere to high standards for research ethics and authorship. Authors of relevant research articles may be asked to complete the questionnaire below, which outlines ethical, cultural, and scientific considerations specific to inclusivity in global research. This questionnaire may be requested when researchers have travelled to a different country to conduct research, if research uses samples collected in another country, research with Indigenous populations or their lands, or if research is on cultural artefacts. Researchers travelling to another country solely to use laboratory equipment will not normally be required to complete the questionnaire. However, the questionnaire can be requested at the journal’s discretion for any submission – if you have been requested to complete this questionnaire by the PLOS journal you submitted to, please do so.

Please complete the questionnaire below and include this as a Supporting Information file with your manuscript. Note that if your paper is accepted for publication, this checklist will be published with your article in the supporting information files. Please ensure that you reference the checklist in the main body of your manuscript. We suggest adding a subsection ‘Inclusivity in global research’ to your Methods section and adding the following sentence: “Additional information regarding the ethical, cultural, and scientific considerations specific to inclusivity in global research is included in the Supporting Information (SX Checklist)”

The questions have been designed to be applicable to a wide range of study types, and there are subsections for both human subjects research and non-human subjects research. If any of the questions are not relevant to your research please mark them as “N/A” as appropriate.

**Ethical considerations, permits and authorship**

*This section is applicable to all research types.*

Provide details as to who granted permissions and/or consent for the study to take place in the Methods section of your manuscript. This should include the names of **all** ethics boards, governmental organizations, community leaders or other bodies that provided approval for the study. If individuals provided approval refer to these people by their role or title but do not list their name(s).

Reported on page number: 12

Ethical approval was obtained from the Research Ethics Review Committee of the World Health Organization (WHO ERC) (protocol ID: ERC.0003259). In Burkina Faso, ethical approval was obtained from the Comité d’Ethique pour la Recherche en santé (CERS), deliberation number 2020-02-030 for the RDS study and no. 2020-02-033 for the qualitative study.

At the community level, we held informational meetings with the Kaya health district officer, and presidents of local women’s and other health associations, who collectively provided permission for all research activities in their communities.

If there were any deviations from the study protocol after approval was obtained please provide details of these changes in the Methods section of your manuscript.

Reported on page number: there was no deviations from the study protocol after approval was obtained

Did this study involve local collaborators that are residents of the country where the research was conducted or members of the community studied? If you do not have any authors from said communities, please provide an explanation for this below.

Four coauthors in this study, including the main author and the last author are residents from Burkina Faso (RC, SK, AB, NVB). Local collaborators were limited to members of the Kaya Health and Demographic Surveillance System (HDSS) team, all of whom reside in the Centre-Nord region of Burkina Faso and are part of the community under study (AB and NVB). This team included the HDSS director (public health scientist), a data-management officer, and field supervisors who conducted household visits and liaised with local health facilities. Each of these collaborators contributed substantially to study conception, design, data collection, analysis, and manuscript drafting, and therefore all meet PLOS’ authorship criteria and are listed as co-authors on the byline. No other external collaborators from the study communities were involved.

Everyone listed as an author should meet PLOS’ criteria for authorship and all individuals who meet these criteria should be included in the author byline, rather than the acknowledgements. For further information please see the journal’s Authorship Policy.

**Human subjects research (e.g. health research, medical research, cross-cultural psychology)**

Did you obtain written informed consent from a representative of the local community or region before the research took place? How did you establish who speaks for the community? Details of written informed consent obtained from study participants should be reported separately in the Methods section of your manuscript.

How did members of the local community provide input on the aims of the research investigation, its methodology, and its anticipated outcome(s)?

When engaging with the local community, how did you ensure that the informed consent documents and other materials could be understood by local stakeholders?

Prior to launching the main RDS survey, we informed the health authorities of the Centre-North region and the Kaya health district, ensuring transparent coordination with local institutions. We conducted a stakeholder mapping exercise and engaged key community-based organizations working in sexual and reproductive health (SRH) to explore local terminology and community perceptions around abortion. This process helped us harmonize and refine our methodological approach.

Key informants identified during this preparatory phase signed informed consent forms, in accordance with ethical standards for research involving human participants. The consent process was adapted to the local context, taking into account literacy levels and cultural sensitivities.

Additionally, our study is embedded within the Health and Demographic Surveillance System (HDSS), coordinated by the IRSS since 2007(**The Kaya HDSS, Burkina Faso: a platform for epidemiological studies and health programme evaluation**

[The Kaya HDSS, Burkina Faso: a platform for epidemiological studies and health programme evaluation - PubMed](https://pubmed.ncbi.nlm.nih.gov/23918849/)

).

The HDSS is well accepted by the local community and involves biannual data collection rounds. Two of our co-authors are currently involved in its management, ensuring deep familiarity with the field and a sustained relationship of trust with the population.

Additionally, our study is embedded within the Health and Demographic Surveillance System (HDSS), coordinated by the IRSS since 2007. The HDSS is well accepted by the local community and involves biannual data collection rounds. Two of our co-authors are currently involved in its management, ensuring deep familiarity with the field and a sustained relationship of trust with the population.

Will the findings of the research be made available in an understandable format to stakeholders in the community where the study was conducted (e.g. via a presentation, summary report, copies of publications, etc.)? Please provide details of how this will be achieved.

The findings of this study will be shared through a restitution workshop to the district health management team, community leaders and all main SRH partners of the health districts. We will also prduce policy briefs to support knowledge sharing. Copies of the publication will also be made available

**Non-human subjects research using specimens/ animals collected as part of the study, or those housed in archival collections. Examples include archaeology, paleontology, botany and zoology.**

Did the permission you obtained from a local authority to perform the study include an agreement on access to outputs and benefit sharing? This may include procedures to enable fair distribution of the benefits and resources arising from the research performed. Please include any details of Prior Informed Consent and Benefit Sharing Agreements obtained. These may be required by field-specific regulations, for example the Convention on Biological Diversity (CBD) and the associated Nagoya Protocol.

N/A

If the material used in your study was imported, please A) provide the year it was imported and B) indicate whether permits were obtained to import/export the materials used, C) provide details of any permits obtained. If this information is not available, please indicate this.

N/A

If you used archival specimens, please state how the material used in your study was acquired by the institute it is held in and provide details of any permits obtained for the original excavations/ sample collection. If this information is not available, please indicate this.

N/A

How was the potential cultural significance of the materials collected in your study to local communities considered in your research design? Were Indigenous peoples and/or local researchers and institutions involved with archaeological excavations / collection of specimens? If so, please provide a description of their involvement.

N/A

If your manuscript includes photographs of human remains please indicate whether authors obtained permission from descendants or affiliated cultural communities to do so.

N/A
