# Supplementary material for: “As it is about that, they do as they please”: Women’s experience of the accessibility and acceptability of postabortion care in Kaya, Burkina Faso
Source: PLOS Glob Public Health. 2026 Jan 8;6(1):e0005035. doi: 10.1371/journal.pgph.0005035 (PMC12782387; doi:10.1371/journal.pgph.0005035)
Supplement: Attachment — Submitted filename: Point by point response to reviewers_PLOS GPH_Jul 2025.docx [file pgph.0005035.s004.docx]

**Point by point response to reviewers’ comments PGPH_Article: “As it is about it”**

Dear Editor and Reviewers,

Thank you for the thorough review of our manuscript. We appreciate you taking the time to provide constructive and valuable feedback to improve our work. Considering your comments, we have carefully revised the abstract to enhance its clarity and ensure that the findings and implications are presented concisely and effectively. Here are our responses to the specific points raised:

**Journal Requirements:**

1. Please provide an Author Summary. This should appear in your manuscript between the Abstract (if applicable) and the Introduction, and should be 150–200 words long. The aim should be to make your findings accessible to a wide audience that includes both scientists and non-scientists. Sample summaries can be found on our website under Submission Guidelines:
https://journals.plos.org/globalpublichealth/s/submission-guidelines#loc-parts-of-a-submission.

As requested, we provided an author summary, lines 55-73, words count: 190, as it appear below:

Author summary

In Burkina Faso, where abortion is legally restricted and socially stigmatized, many women face significant challenges when seeking post-abortion care (PAC). Our study, conducted in Kaya, a semi-urban secondary city, examined how different women experience and access these essential health services, particularly following abortion-related complications. Through detailed interviews with 35 women who sought PAC and eight representatives from grassroots sexual and reproductive health organizations, we uncovered striking disparities in care access and quality based on women's social circumstances. Notably, unmarried women, especially adolescents, encounter severe stigma, including verbal abuse, and extended waiting times in public healthcare facilities. These women faced substantial stigma, leading to delayed care and negative experiences, including verbal abuse and lack of privacy in public facilities. By contrast, married and wealthier women generally received better treatment, particularly those who could access private facilities. Our study highlights the urgent need for healthcare interventions to address abortion stigma and the resulting inequalities in PAC. This includes improving provider attitudes, strengthening the healthcare infrastructure, and promoting community awareness of legal exceptions to abortion restrictions. Ultimately, equitable and patient-centred PAC is crucial for improving women's health and achieving broader health equity.

2. In the online submission form, you indicated that "The data supporting the findings of this study are available from the corresponding author upon reasonable request.".

All PLOS journals now require all data underlying the findings described in their manuscript to be freely available to other researchers, either 
a. In a public repository, 
b. Within the manuscript itself, or 
c. Uploaded as supplementary information.

This policy applies to all data except where public deposition would breach compliance with the protocol approved by your research ethics board. If your data cannot be made publicly available for ethical or legal reasons (e.g., public availability would compromise patient privacy), please explain your reasons by return email and your exemption request will be escalated to the editor for approval. Your exemption request will be handled independently and will not hold up the peer review process, but will need to be resolved should your manuscript be accepted for publication. One of the Editorial team will then be in touch if there are any issues.

Thank you for this precision. Due to the sensible nature of the data and in compliance with our protocol that was approved by our local research ethics board, we are afraid we cannot openly share the data for this study for confidentiality reasons. As suggested, we will get in touch with the Editorial team to handle this issue.

**Reviewer #1:**

Abstract

1. • Clarify what types of associations the representatives are from. Are they activism groups, medical professionals, grassroots organizations, governmental bodies, or other entities?

Line 29-33: “These women sought PAC in formal healthcare services due to abortion-related complications. Additionally, interviews were conducted with representatives from associations which are grassroot organizations actively engaged in sexual and reproductive health in town (n=8).”

Introduction

2. • Line 119-124: “Approximately 90% of abortions are unsafe, and there is evidence of disparities among older women, women with less education, and women living in rural areas [31]… However, despite these policies, almost 40% of women who experience abortion-related complications do not receive the necessary medical care [31].” Presenting these data, which are from Argentina, Bangladesh, Ethiopia, and Nigeria, might mislead readers into thinking that the data belongs to Burkina Faso. Do you have local data available

We agree that this can be misleading, as the data initially referenced in this section are from studies conducted in Argentina, Bangladesh, Ethiopia, and Nigeria. We revised the manuscript to include equivalent national-level data for these measures, from the Performance monitoring for action (PMA 2020) study in Burkina Faso (2020-2021). We have clarified this distinction in the revised manuscript to avoid any potential confusion for the readers. The data now reads as follows in the manuscript in lines 139-145:

“According to the PMA abortion survey results in the country in 2022, over half of abortions were most unsafe (51%) of abortions are unsafe, and there is evidence of disparities among older women, women with less education, and women living in rural areas[17]. PAC is an integral part of the national free care policy and falls under the Emergency Obstetric and Newborn Care (EmONC) framework [18]. However, despite these policies, only half of the women who reported a potential severe abortion complication accessed PAC at a facility for treatment[17].“

3. • Line 141: “Our data collection tool incorporated questions from both the WHO framework for quality of care [22] and the abortion stigma literature.” Consider adding a citation for the abortion stigma literature.

Thank you for your relevant comment. We added the citations of the references used in our conceptualization in the manuscript as requested. This appears now in the manuscript as follows in lines 162-163:

“Our data collection tool incorporated questions from both the WHO framework for quality of care [23] and the abortion stigma literature[12,24–26]. ”

4. • Line 150: “Contributing to the achievement of sustainable development goals (SDGs).” It would be helpful to specify which SDGs your study contributes to.

We appreciate and we agree with your suggestion for specificity. In the revised manuscript, we have clarified that in our study as follows in lines 168-173:

“Our research hypothesized that understanding the cultural context and client perspectives is crucial for improving the accessibility and acceptability/person centeredness of PAC. This approach supports equitable access to quality care for abortion complications in Burkina Faso’s restrictive setting, contributing to SDG 3.7 and SDG 5.6 on universal access to sexual and reproductive health services and rights [27].”

Materials and Methods

5. Line 160-166: “The Kaya health district includes forty (40) first-level public health facilities, including 39 primary health and social promotion centers (CSPSs) and one medical center with a surgery unit (CMA). In addition, there were four private and confessional health facilities. The health district includes the regional hospital of Kaya (CHR), which is a referral hospital for the Centre-north region. In 2020, 53 obstetric complications due to unsafe abortions were reported in the Kaya Health District, representing 8.3% of all obstetric complications (in the country) [16].” What are the capacities of these health facilities? Do they all provide PAC?

We appreciate your question regarding the capacities of the health facilities and their ability to provide Postabortion Care (PAC). The Kaya Health District comprises 40 primary health facilities, one medical center with a surgical unit (CMA), and four private/confessional health facilities. PAC services are primarily concentrated in the CMA and the regional hospital of Kaya (CHR), which also functions as a referral hospital. While some of the primary health and social promotion centers (CSPSs) offer basic care, comprehensive PAC services, including the management of abortion complications, are limited to higher-level facilities like the CMA and CHR.This differentiation in capacity levels, where the CMA and CHR provide more comprehensive PAC services compared to the primary health facilities, has been clarified in the revised manuscript (lines 182-190):

“The Kaya health district includes forty (40) first-level public health facilities, including 39 primary health and social promotion centers (CSPSs) and one medical center with a surgery unit (CMA). In addition, there were four private and confessional health facilities. The health district includes the regional hospital of Kaya (CHR), which is a referral hospital for the Centre-north region. While some of the CSPSs offer basic care, comprehensive PAC services, including the management of abortion complications, are primarily concentrated to higher-level facilities like the CMA and CHR. In 2020, 53 obstetric complications due to unsafe abortions were reported in the Kaya Health District, representing 8.3% of all obstetric complications [16].”

6. I would like to know more about how the interview guides overtime based on the emerging themes. If possible, please detail the process and present your different versions of question guides and final codebook for both types of participants.

Thank you for your thoughtful request. The guides were initially developed based on the literature and the conceptual frameworks (the Interpersonal Quality in Abortion Care scale, the WHO quality of care framework, etc.). Initial questions focused broadly on abortion experiences, care-seeking behaviours, and perceptions of health services. the initial guides were developed iteratively in response to insights arising from early interviews and thematic reflections conducted by the research team. As interviews progressed, the guides were adapted to explore emerging areas such as social stigma, legal awareness, facility-level discretion, and expectations of dignity in PAC.

We conducted informal debriefings with fieldworkers after each round of interviews, which helped refine prompts and improve the cultural and emotional sensitivity of our wording. For instance, questions regarding abortion motives were rephrased to avoid judgmental framing, while new probes were added to explore patients’ knowledge of the law and their interactions with providers more deeply. Final versions of the guides included tailored pathways depending on participant type (women with PAC experience vs. association representatives), ensuring questions were adapted to respondents' roles, experiences, and comfort levels.

We described this process in the data collection sub section.

7. • Include the length of the interviews in the Results section.

The lengths of interviews are already included in the methods section. Interviews with women lasted between 30 minutes and 2 hours, while interviews with association representatives typically lasted between 1 and 2 hours. We amended the data in the methods section to be more precise as follows (lines 233-234): “The interviews lasted between 30 minutes and two hours.” And lines 256-257: “These interviews lasted between one and two hours and were recorded with the consent of the respondents.”

We think that it will be a repetition to include the same information in the results section. However, we will comply with your request if it enhances the clarity of the manuscript.

8. • Regarding the note-taking process during interviews: Since voice recording was not possible due to sensitivity concerns, did researchers take real-time transcripts in English, Moore, or French? Were the transcripts and field notes written simultaneously? Did the interviewers reflect on the interviews afterward? Was there a translation process involved? Who coded the transcripts, and what are their language proficiencies? Were they acknowledged in the author list?

We thank you for this thoughtful comment. We have clarified the note-taking and transcription process in the revised manuscript. While all research assistants are fluent in both Moore and French, they could not write in Moore. Real-time notes were taken primarily in French, with occasional use of Moore ( Moore expressions transcribed in French) to capture culturally specific expressions. In most cases, field notes and transcripts were written simultaneously during the interview. Immediately after each interview, the research assistants expanded their notes to ensure completeness and to integrate contextual and nonverbal observations. These expanded transcripts were then translated into French when necessary, preserving the original meaning and cultural nuances.

All transcripts were coded in French by the main author who is also fluent in both languages. The fieldworkers are not cited as co-authors; although they made essential contributions to data collection, they were not involved in the analysis, interpretation, or manuscript development. While the assistants are not listed as co-authors, they are acknowledged in the manuscript for their contributions to data collection in the Acknowledgement section.

We have added details on the language used during note-taking, the process of simultaneous and post-interview transcription, the translation workflow, and the language proficiency of the coding team (see lines 230-242 and 251-259 in the data collection and treatment sub-section).

9. • Clarify whether each participant was interviewed by a single researcher or if additional personnel assisted with field notes. Recognize the limitations of your methods in transcribing, note-taking, and translation.

We appreciate this important point. Each participant was interviewed by a single research assistant, with no additional personnel present. This approach was chosen to ensure privacy and participant comfort, given the sensitivity of the topic. We have now explicitly stated this in the manuscript (see lines 231-233).

We also acknowledge the methodological limitations associated with real-time note-taking in the absence of audio recordings. While this may have limited the verbatim capture of participant narratives, we mitigated this by expanding notes immediately after interviews and through bilingual triangulation during translation and coding. These limitations and mitigation strategies are now discussed in the revised manuscript (lines 843-847).

10. • Elaborate on whether the authors discussed findings with the data collection and coding teams. How were discrepancies among coders resolved? What was the level of agreement between coders?

We thank you for raising this important aspect of qualitative rigor. Throughout the study, the lead author held regular debriefing sessions with the research assistants to reflect on interview dynamics, ensure the completeness of the interviews and of the different themes, identify emerging themes, and adapt the interview guide. During the coding phase, the lead author coded all transcripts, using the Braun and Clarke’s reflective thematic analysis approach. This approach does not require multiple coders or formal inter-coder reliability metrics, as it prioritizes the researcher’s interpretive engagement with the data. The lead author, who is bilingual in Moore and French, conducted all coding and theme development, ensuring linguistic and cultural sensitivity. Reflexivity was maintained through analytic of memos and iterative review of transcripts. While coding was not duplicated, the lead author held regular debriefings with the data collection team and also discussed the core research team who is very familiar to the context, to discuss emerging interpretations and ensure contextual fidelity. These collaborative reflections helped enrich the analysis and mitigate limitations associated with single-coder approaches.

We have revised the manuscript accordingly, and it now reads as follows in lines 267-273 and 294-303. We also added a paragraph on the limitation of this approach in the discussion section in lines 853-857.

11. • While you attached the SRQR, there are still ambiguities in your data analysis methods. Please provide further detailed clarification.

While the SRQR checklist was included as supplementary material, we recognize that further detail on the data analysis process was needed. We restructured all the data analysis subsection for more clarity. This section now appears in the manuscript as follows (lines 267-310):

“We conducted thematic analysis using Braun and Clarke’s reflexive approach, which emphasizes the researcher’s active role in interpreting patterns of meaning within qualitative data. This process involved six iterative phases: familiarization, initial coding, theme development, theme refinement, theme definition, and final reporting. The lead author, fluent in both Mooré and French, performed all coding in French, drawing on her bilingual and contextual expertise to ensure culturally grounded interpretations.

The initial codebook was informed by existing literature on abortion stigma and patient experience in abortion and PAC contexts [12,13,23,32–34], with additional inductive codes developed as themes emerged from the dataset. The analysis focused on two core dimensions of the WHO’s Quality of Care framework: accessibility and acceptability/patient-centeredness (Fig 1) [23]. Accessibility refers to timely, geographically reasonable, and affordable provision of care. All inputs should be accessible within a safe physical reach for all populations, with an emergency care function of 24 h/7 days and free or affordable health services based on non-discrimination and equity. Acceptability or patient-centeredness includes patient preferences and aspirations that should be considered in the healthcare provided. Care should be patient-centered, respectful of the patient’s values and choices to promote patient satisfaction and fulfillment of human rights. Moreover, the environment should be safe, clean, appealing, and designed to respect the privacy and confidentiality of those seeking these services. Healthcare providers should be non-judgmental, considerate, and easy to relate. The facility ensures that a system for triage is in place, and that consultations occur within a short waiting time, with or without an appointment, and (where necessary) swift referral is organized [23]. Abortion stigma was assessed through respondents’ perceptions of community attitudes toward this practice, their knowledge of the law, and how women view their own abortions in view of prevailing social expectations.

Interpretive depth was enhanced through regular debriefings between the lead author and the research team that supervised the RDS study, who shared reflections from fieldwork that shaped thematic focus and contextual alignment. Additionally, peer consultations with co-authors supported critique and refinement of thematic categories throughout the analysis.

Although coding was conducted by a single researcher, reflexivity was rigorously maintained through analytic memoing, ongoing documentation of interpretive decisions, and structured reflection on the author’s positionality. These reflexive practices, alongside collaborative review of emerging findings, mitigated limitations associated with single-coder analysis and strengthened the study’s trustworthiness.

To further link macro-level cultural norms with individual care experiences, we developed two typologies based on women’s socioeconomic status and the degree of stigma surrounding their abortion. These typologies provided a framework for analyzing variation in PAC trajectories and barriers to acceptable care. All data were analyzed using ATLAS.ti software (version 8.4.26.0, 2019).

This study adhered to the Standard for Reporting Qualitative Research (SRQR) [30], that is provided as supplementary file (S1 File).”

Ethical Considerations

12. Since women below 18 were included, how was their consent obtained—through parental consent or another method?

Thank you for highlighting this point. In accordance with the approved protocol by the national ethics committee, verbal informed consent was obtained directly from all participants aged 15 and older. For participants under the age of 15, parental consent was first obtained before seeking the participant’s assent to ensure ethical inclusion and respect for autonomy. To protect participants and minimize potential social exposure or discomfort, questions related to abortion were embedded within a broader thematic focus on sexual and reproductive health. We amended the Ethical Considerations section as follows (lines 311-320):

“This study was approved by the National Ethics Committee for Research in Health of Burkina Faso (no. 2020-02-033). Participation in the study was voluntary. Verbal informed consent was obtained from each respondent before conducting the interviews. For participants under the age of 15, parental consent was obtained before seeking verbal assent from the participant. This two-step procedure ensured compliance with national ethical regulations and safeguarded the rights of minors participating in research related to sensitive topics. Anonymity and confidentiality were guaranteed in the analysis and subsequent reporting of the study results by removing all identifiers or personal information.”

13. • Why was only verbal informed consent obtained? Were there risks associated with obtaining written consent? What measures were taken to ensure participants understood the risks involved in the study?

Verbal informed consent was used to minimize potential risks to participants in a sensitive legal and sociocultural context. Given the highly stigmatized nature of abortion in Burkina Faso and its legal restrictions, requesting written consent could have generated discomfort, raised confidentiality concerns, or deterred participation, especially among women seeking care outside formal channels. This approach was approved by the national ethics committee for research in health of Burkina Faso first, for the initial main RDS study, and then for this current study. This approach aligned with ethical recommendations for research in settings where signed documents may inadvertently compromise anonymity or be perceived as linked to legal authority.

To ensure participants fully understood the study and the risks involved, the consent process included a clear oral explanation of:

- The study’s purpose and voluntary nature
- The right to decline participation or withdraw at any time
- Measures in place to guarantee anonymity and confidentiality
- How data would be recorded (without voice recording for interviews with women) and used

The research assistants, trained in ethics and fluent in the local languages (Moore and French), used culturally appropriate and accessible language throughout the consent process. Ample time was given for participants to ask questions before giving consent.

Results

14. • Ensure consistency in how participant information is presented, including age, gender, marital status, and professional titles.

Thank you for this observation. We have revised the Results section to ensure consistency in presenting participant information, including age, gender, marital status, and professional titles. Participant data was harmonized across the section for clarity and uniformity.

15. • Consider moving the section on “sociodemographic characteristics of abortion seekers and reasons for abortion” and Table 1 to the beginning of the Results section. This reorganization would provide a clearer overview of the participants and improve the flow of your analysis.

Following your suggestion, we have moved this section and Table 1 to the beginning of the Results section to provide a clearer overview of the participants, as suggested. This reorganization improves the flow of the section and enables readers to better understand the context of the subsequent analyses.

16. • Line 483: “As it is about that, they do as they please”—This phrase appears in both the title and subtitle but is somewhat ambiguous. It was unclear initially whether it represents the perspective of the women or the care providers. In the Results section, this phrase is linked to the acceptability/person-centeredness sub-section but is only discussed later under “Patient satisfaction and fulfillment of human rights.” Consider clarifying how you structured the Results section and whether this quote should be emphasized as a key finding.

Thank you for this insightful comment. The phrase “As it is about that, they do as they please” is a direct quote from a participant, reflecting her perception, and a general perception among the participants in our study, of the arbitrariness and lack of patient-centeredness in provider behavior during post-abortion care. It captures a critical dimension of dissatisfaction and disempowerment among young women who seek PAC in public facilities, where stigma influences provider attitudes and discretion.

To improve clarity, we have restructured the whole subsection (lines 539-618) and supports our interpretation of PAC experiences as shaped by stigma, discretion, and unmet expectations of respectful care, by:

- reframed the section heading to better reflect the quote’s origin and its link to patient perceptions
- relocated and highlighted the quote earlier in the acceptability/person-centeredness subsection, where it thematically belongs
- clarified in the text that the quote represents the perspective of the participants, not the providers themselves.

17. • Since your analysis compares perspectives from younger single women and older married women, creating a table to summarize the differences and similarities between these groups would greatly enhance clarity. It will be great if you can also visualize your findings by creating a conceptual diagrams.

We thank you for this suggestion. A new table has been created to summarize the differences and similarities between the two demographic groups (younger single women and older married women) regarding their experiences and reasons for abortion (lines 541-545 , table2). Additionally, we have included a conceptual diagram to visualize the findings, providing a clear depiction of the relationships between sociodemographic factors, abortion motives, and care access pathways (lines 715-719, fig 2).

Discussion

18. • After introducing the abbreviation for PAC, avoid repeating “postabortion care (PAC)”.

Thank you for this observation. We have carefully revised the document to avoid repeating “postabortion care” beyond its first introduction. We now consistently use the abbreviation “PAC” in the Results section and elsewhere in the manuscript.

19. • Line 622: Consider rephrasing “Clients” for clarity.

We agree with the reviewer that the term “Clients” may be unclear. We have revised it to “women seeking PAC” throughout the manuscript to provide clarity and ensure consistency in terminology.

20. • Line 736-747: Expand on the study's implications for both patients and communities.

Thank you for this suggestion. We have expanded this section to elaborate on the broader implications of our findings. Specifically, we discuss how addressing the inequalities identified in PAC provision can improve health outcomes for women, particularly unmarried and socioeconomically disadvantaged women. We highlight the need for community interventions to reduce abortion stigma and structural barriers, which could lead to more equitable access to care and better community understanding of the challenges faced by women seeking PAC. The paragraph now reads as follows, lines 811-835:

“Our findings offer valuable insights for policymakers, mid-level managers, healthcare providers, and researchers in designing, monitoring, or assessing the implementation of safe abortion or PAC policies. The narratives of women regarding their experiences in accessing PAC in Kaya highlights the significant influence of stigma, social identity, and provider discretion on both their expectations and experiences of care. This is particularly evident among adolescents and young unmarried women. Many of these individuals expressed relief at avoiding humiliation or legal repercussions rather than satisfaction with the quality of care itself, thereby highlighting diminished expectations that reflect broader systemic inequities. To ensure equitable access and person-centered treatment, PAC strategies must be implemented at multiple levels. National health policies should promote consistent standards of respectful and rights-based care to enhance the quality of PAC, particularly in Kaya and similar settings, and more broadly across the entire country. This approach aims to prevent variations in care quality based on marital status, socioeconomic background, or geographic location. Furthermore, improvements at the facility level, including adequate infrastructure, privacy, and youth-sensitive provider training, are crucial for increasing acceptability and restoring patient agency. At the community level, limited awareness of abortion laws and strong moral condemnation continue to delay care-seeking and reinforce stigma. Enhancing public education initiatives on reproductive rights and the availability of PAC, as well as engaging trusted local actors to promote open dialogue, can contribute to altering detrimental narratives and improving community support for PAC services. Lastly, targeted strategies for adolescents and young people are essential. Strengthening youth-friendly services and integrating comprehensive sexuality education into school curricula can help improve knowledge, reduce stigma, and facilitate timely and dignified access to care.”

21. • Acknowledge the limitations of your transcribing, note-taking, and translation methods, as noted earlier.

We appreciate this observation and have included an acknowledgment of these limitations in the revised Discussion section. Specifically, we note that the transcription process, conducted from audio to text, may have led to some loss of nuance in participants' expressions. Furthermore, note-taking during interviews might introduce omissions, and translations from local languages to French and English could inadvertently lose some cultural context or specific meanings. We have acknowledged these limitations in lines 842-853:

“ Given the sensitive nature of the topic and participants’ refusal to be recorded, data collection relied on real-time note-taking during interviews. While interviewers expanded field notes immediately afterward to ensure completeness and context, this method carries inherent risks, including incomplete transcription and reduced verbatim accuracy. We also acknowledge the potential for desirability bias, as abortion remains socially disapproved in the study setting. Some respondents may have shaped their narratives to conform to normative expectations, despite assurances of confidentiality. Furthermore, translation from the local language to French first, and then into English may have resulted in the loss of linguistic nuance, despite the lead author’s bilingual proficiency and collaborative debriefings with the research team. These limitations may have influenced the precision of participant accounts and thematic interpretation.”

22. • Since this is a qualitative study with a relatively small sample size, dividing women into specific age bucket and marital status categories might introduce bias. Acknowledge this limitation and avoid using potentially causal languages throughout the article.

Thank you for this important observation. We have revised the limitations section to acknowledge this issue and clarify that the groupings were primarily used to explore patterns emerging from participant narratives, without assuming causality. We have also reviewed the manuscript to ensure that causal language is avoided, and interpretations are presented with appropriate caution. Lines 857-863:

“Finally, we acknowledge that dividing participants into specific age groups and marital status categories may introduce bias, especially given the limited sample size and qualitative nature of the study. These classifications were used descriptively to examine recurring patterns in women's narratives but do not reflect rigid or causal associations. Experiences of stigma, access, and provider interactions are inherently nuanced, and we caution against generalizing these groupings beyond the context of this study.”

**Reviewer #2:**

The abstract explores the challenges faced by women in accessing postabortion care (PAC) in Kaya, Burkina Faso, emphasizing the impact of legal restrictions and social stigma, particularly for impoverished rural women. Using in-depth interviews with 35 women and 8 association representatives, the study identifies inequalities in PAC access and acceptability, shaped by women’s sociodemographic status. Findings reveal that abortion stigma is stronger for unmarried women, delaying care-seeking and leading to verbal abuse, longer waits, and lack of privacy, whereas married women face less stigma due to socially accepted reasons for abortions. Socioeconomic disparities also influence care, with wealthier women accessing better-quality private facilities. The study highlights significant inequalities and calls attention to the need for policies addressing the stigma, prosecution fears, and disparities in PAC provision.

Thank you for your comment. We appreciate your detailed summary of the abstract and the insights you have provided. As this summary accurately represents our work, we have not made any modifications to the abstract in response to your review. We appreciate your careful reading and comprehensive understanding of our research.
