# Supplementary material for: “As it is about that, they do as they please”: Women’s experience of the accessibility and acceptability of postabortion care in Kaya, Burkina Faso
Source: PLOS Glob Public Health. 2026 Jan 8;6(1):e0005035. doi: 10.1371/journal.pgph.0005035 (PMC12782387; doi:10.1371/journal.pgph.0005035)
Supplement: Attachment — Submitted filename: Response to Reviewers.docx [file pgph.0005035.s006.docx]

**Response to Reviewers**

**Manuscript ID:** PGPH-D-24-02701R2

**Title:** *"As it is about that, they do as they please":* Women’s experience of the accessibility and acceptability of postabortion care in Kaya, Burkina Faso

**General Response to Editor and Reviewer**

We would like to sincerely thank the editor and reviewer for their thoughtful evaluation of our manuscript and for the constructive comments provided. We are grateful that the reviewer found the revision strong and nearly ready for publication. We have carefully addressed the remaining minor issues, including clarifying the distinction between “informal” and “formal” professions in Table 1 and harmonizing the wording of the “number of children” variable across the table and text.

We reviewed our reference list to ensure that it is complete and correct. No paper was retracted. There were no changes to the reference list.

We have also carefully read and thoroughly edited the manuscript, Minor typographical and formatting corrections have been made throughout the manuscript to ensure consistency, clarity, and compliance with journal requirements before resubmission.

Figures have been processed through the PLOS NAAS tool to ensure compliance with submission requirements.

We appreciate the reviewer’s concern regarding data availability. We would like to note that we had previously communicated with the journal office to clarify this point, and the Data Availability Statement has been thoroughly explained in the dedicated section of the submission platform. In line with PLOS requirements, we specified that anonymized transcripts and coding frameworks are available, while raw interview notes cannot be shared publicly due to confidentiality restrictions. We previously provided all documents required for this justification, and edited the Data availability statement accordingly. Access conditions for qualified researchers have also been detailed in the submission system.

We believe these revisions have further strengthened the clarity and consistency of our manuscript.

**Reviewer #1 Comments and Author Responses**

**Comment 1:** *In Table 1, it would help to clarify the distinction between “informal” and “formal” professions—this could be addressed with a brief footnote.*

**Response:** We appreciate this suggestion. We have added a footnote to Table 1 clarifying that:

- *“Informal” refers to occupations without formal contracts or social protection (e.g., petty trade, domestic work, casual labor).*
- *“Formal” refers to occupations with formal contracts and social protection (e.g., salaried employment in government or private institutions).*

This clarification ensures readers understand the distinction.

**Comment 2:** *In Table 1, the “number of children” category lists 0, whereas page 16 lists under socio-demographics; this appears to be an inconsistency that can be easily corrected.*

**Response:** Thank you for noting this inconsistency. The issue was related to how the table header repeated when the table extended across two pages. We have corrected this by adjusting the cell properties in the table formatting so that the header now displays consistently. In addition, for clarity and consistency across the manuscript, we have replaced “1 and +” with “≥ 1” in both the table and the text. We have corrected this inconsistency by adjusting the table formatting.

These changes ensure clarity and consistency in the presentation of sociodemographic characteristics.

We thank the reviewer and editor once again for their constructive feedback and guidance throughout the review process. We believe that the revisions made have strengthened the clarity, consistency, and transparency of our manuscript. We hope that the revised version will now be suitable for publication in *PLOS Global Public Health*.
